# Clinical and radiological features of malformed mesiodens in the nasopalatine canal: an observational study

**DOI:** 10.1093/dmfr/twae003

**Published:** 2024-01-24

**Authors:** Yu-Ri Kim, Yu-Min Lee, Kyung-Hoe Huh, Won-Jin Yi, Min-Suk Heo, Sam-Sun Lee, Jo-Eun Kim

**Affiliations:** Department of Oral and Maxillofacial Radiology, School of Dentistry and Dental Research Institute, Seoul National University, Seoul, 03080, Korea; Department of Oral and Maxillofacial Radiology, School of Dentistry and Dental Research Institute, Seoul National University, Seoul, 03080, Korea; Department of Oral and Maxillofacial Radiology, School of Dentistry and Dental Research Institute, Seoul National University, Seoul, 03080, Korea; Department of Oral and Maxillofacial Radiology, School of Dentistry and Dental Research Institute, Seoul National University, Seoul, 03080, Korea; Department of Oral and Maxillofacial Radiology, School of Dentistry and Dental Research Institute, Seoul National University, Seoul, 03080, Korea; Department of Oral and Maxillofacial Radiology, School of Dentistry and Dental Research Institute, Seoul National University, Seoul, 03080, Korea; Department of Oral and Maxillofacial Radiology, School of Dentistry and Dental Research Institute, Seoul National University, Seoul, 03080, Korea

**Keywords:** mesiodens, nasopalatine canal, morphology, cone-beam computed tomography, complication

## Abstract

**Objectives:**

The purpose of this study is to investigate the morphological changes that occur when mesiodens is located within the nasopalatine canal, as well as clinical characteristics.

**Methods:**

Clinical records and CT images of patients who had mesiodens in the nasopalatine canal were retrospectively analysed. In addition to demographic information, clinical symptoms and complications associated with extraction of mesiodens were recorded. Using CT images, number, location, size, and tooth morphology were evaluated.

**Results:**

This study included 32 patients and 38 mesiodens within the nasopalatine canal. Supernumerary teeth exhibited a characteristic feature of thin and elongated shape in the canal (narrow width and elongation were observed in 96.6% and 53.3% of the patients, respectively). Fusion was found in 4 patients and dilaceration in 12. A complication occurred in 2 patients, which was tooth remnant, not a neurologic complication. Only 5 mesiodens could be detected in the nasopalatine canal on panoramic images.

**Conclusions:**

Morphological abnormalities in mesiodens within the nasopalatine canal were frequently detected, and these could be effectively diagnosed through 3D imaging analysis.

## Introduction

A mesiodens is a supernumerary tooth located between the maxillary central incisors. While the global occurrence rate of mesiodens ranges between 0.15% and 1.9%,^1–4^ it is notably higher in the sub-Saharan and Asian demographics, with estimates hovering between 2.7% and 3.4%.^5^ Mesiodens can cause various complications, from primary teeth retention and delayed eruption to more severe issues, including tooth resorption, pulp necrosis, nasal eruptions, and even dentigerous cyst formations.^6–9^ However, the question of when to surgically remove mesiodens remains controversial. Some authors argued against removal unless the mesiodens obstruct permanent tooth eruption or exhibit pathological signs.^10^ However, some literature suggests that early removal, particularly during the mixed dentition phase (before age 10), can facilitate spontaneous permanent tooth eruption.^11–14^ If mesiodens extraction is indicated, the latest consensus suggests that age should not be a deterrent to prompt removal.^15^

Radiographic imaging has become pivotal in the identification of supernumerary tooth position, number, and morphology. Traditional imaging modalities for mesiodens evaluation include panoramic, periapical, and occlusal radiographs. Although these two-dimensional imaging modalities are common, they cannot find the three-dimensional association between supernumerary teeth and neighbouring structures, such as roots and the nasopalatine canal. Hence, for those opting for extraction, three-dimensional analysis via multidetector row CT (MDCT) or cone-beam CT (CBCT) is preferred. A three-dimensional evaluation is particularly necessary when the surgeon needs to understand the association among vital structures, such as the nasopalatine canal including the nerve and blood vessels. Most of the previous studies have focused on morphological analysis of mesiodens;^10^^,^^15^^,^^16^ however, there were only few case reports on mesiodens that showed nasopalatine canal encroachment. Furthermore, no study focused on the detailed morphological characteristic of mesiodens within the nasopalatine canal.

We hypothesized that when mesiodens is located within the nasopalatine canal, it may exhibit morphological variations influenced by the canal’s shape; thus, extraction of such mesiodens could result in complications. Therefore, this study aimed to analyse the clinical and three-dimensional imaging features of mesiodens in the nasopalatine canal.

## Methods

### Patient selection

**Figure 1.**
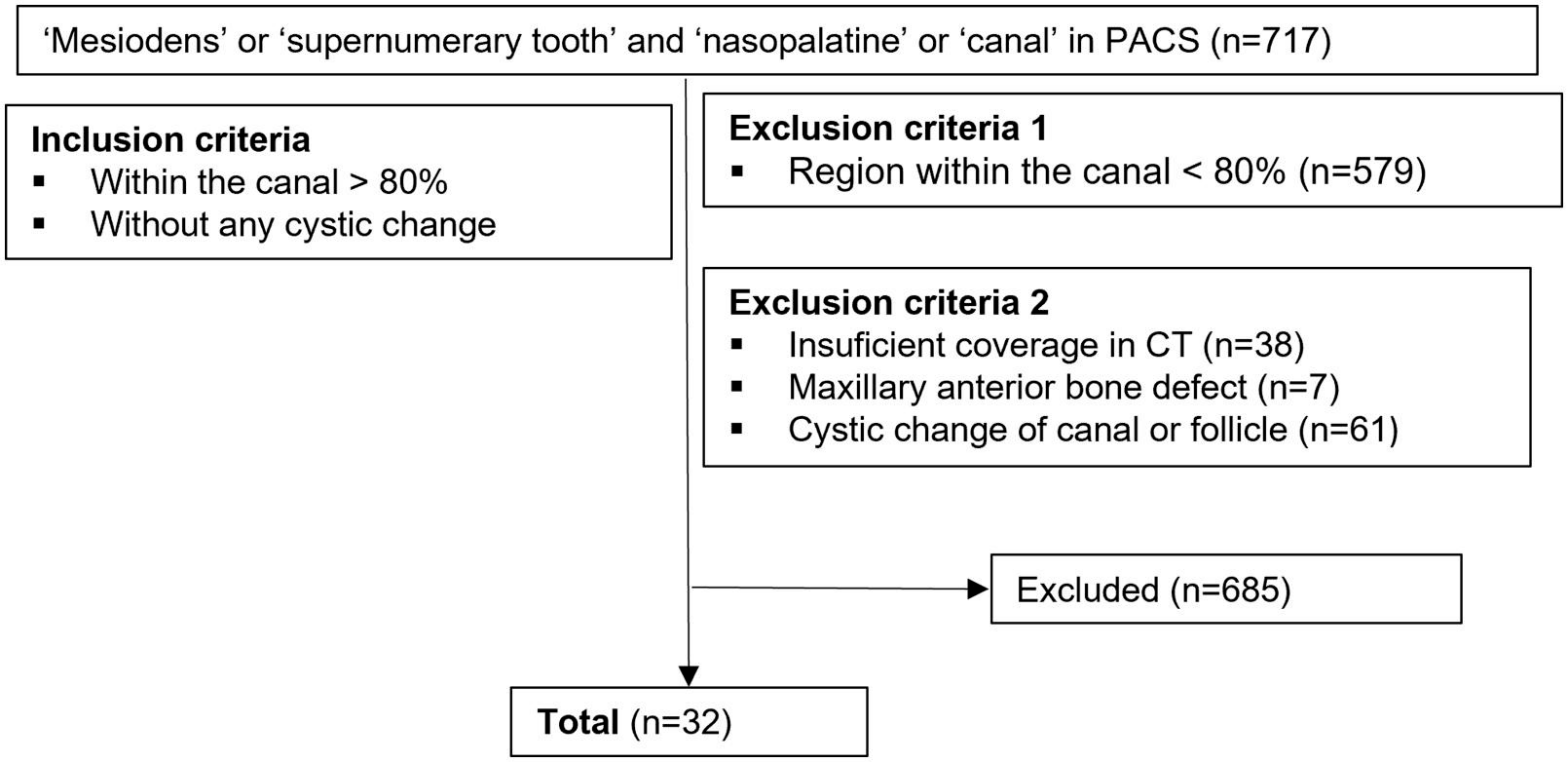
Flowchart of patient selection.

This study was approved by the Institutional Review Board of Seoul National University Dental Hospital (SNUDH) [IRB No. ERI23043]. The requirement of patient consent was waived due to the retrospective nature of the study. We retrospectively reviewed CT or CBCT data of patients who visited SNUDH between January 2000 and August 2023. We used the keyword “supernumerary tooth” or “mesiodens” in conjunction with “nasopalatine” or “canal” in the search within the conclusion section of radiology reports using the Picture Archiving and Communications System (INFINITT PACS, INFINITT Healthcare, Seoul, South Korea). Patients with mesiodens where over 80% of the tooth volume was situated within the nasopalatine canal were included in the study. On the other hand, patients whose mesiodens only marginally invaded the nasopalatine canal or the canal’s cortex externally were excluded. Patients with potential signs of nasopalatine canal cysts or dentigerous cysts of mesiodens were also excluded. The cases that did not sufficiently encompass the region of interest for three-dimensional evaluation and those with maxillary anterior bone defects due to cleft were excluded. Of the 717 selected patients, 32 who met the criteria were included in the final analysis (Figure 1).

### Clinical data collection

Patients’ demographic and clinical data were obtained from electronic dental records. Clinical features such as chief complaints, past medical and dental histories, current illness, clinical diagnoses, treatments, and prognosis were assessed. This included evaluation of complications associated with extractions.

### Image analysis

From the dataset, 32 CT images (15 MDCT, 17 CBCT images) were analysed. Radiological evaluations were performed by consensus of two experienced oral and maxillofacial radiologists with clinical experience of over 10 years.

**Figure 2.**
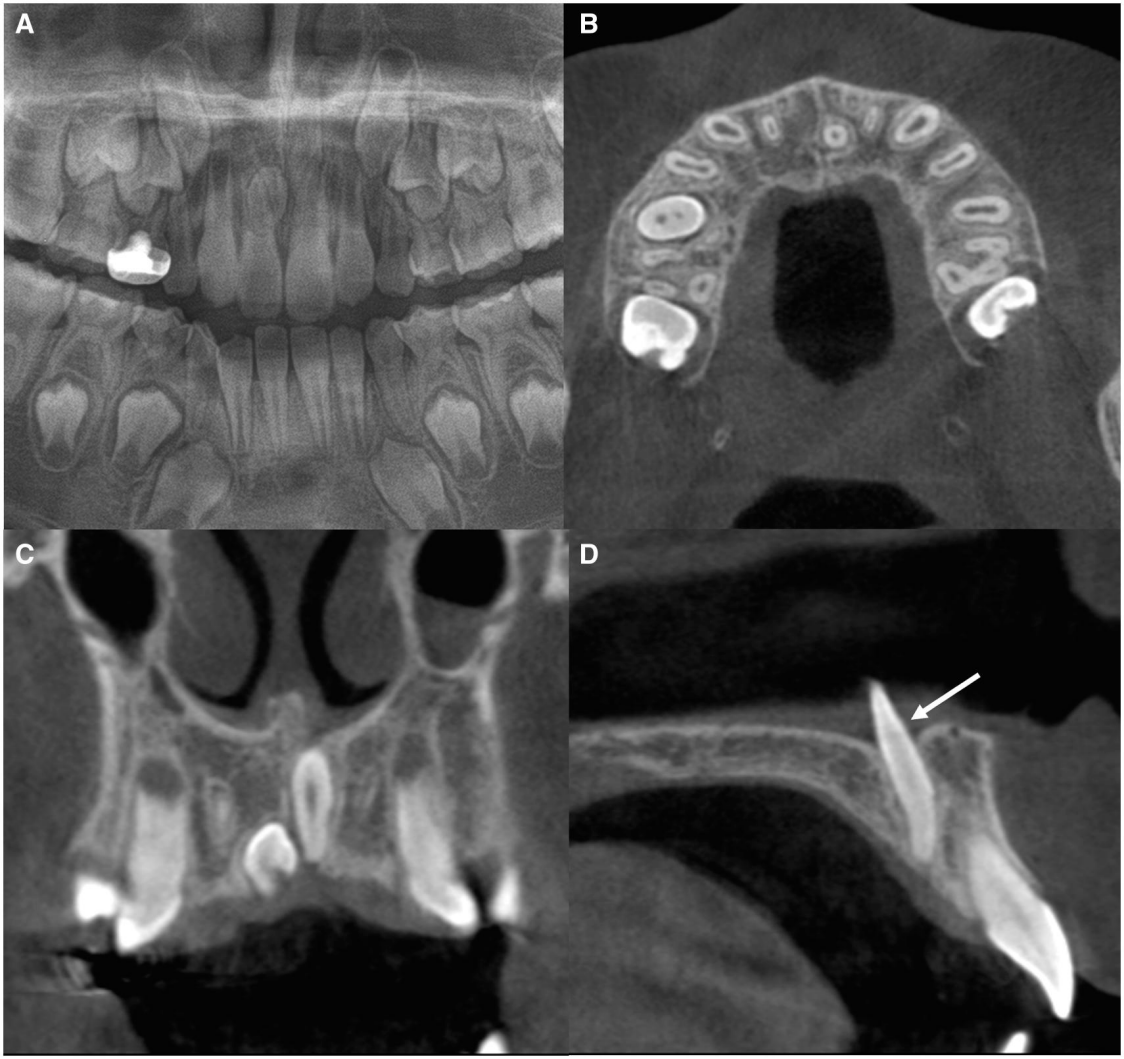
Example of elongated mesiodens in case 13. (A) Cropped panoramic radiograph. (B-D) Cropped CBCT images (axial, coronal, and sagittal views, respectively). Note the elongation in the crown portion of mesiodens within the nasopalatine canal and nasal cavity floor (D, arrow).

**Figure 3.**
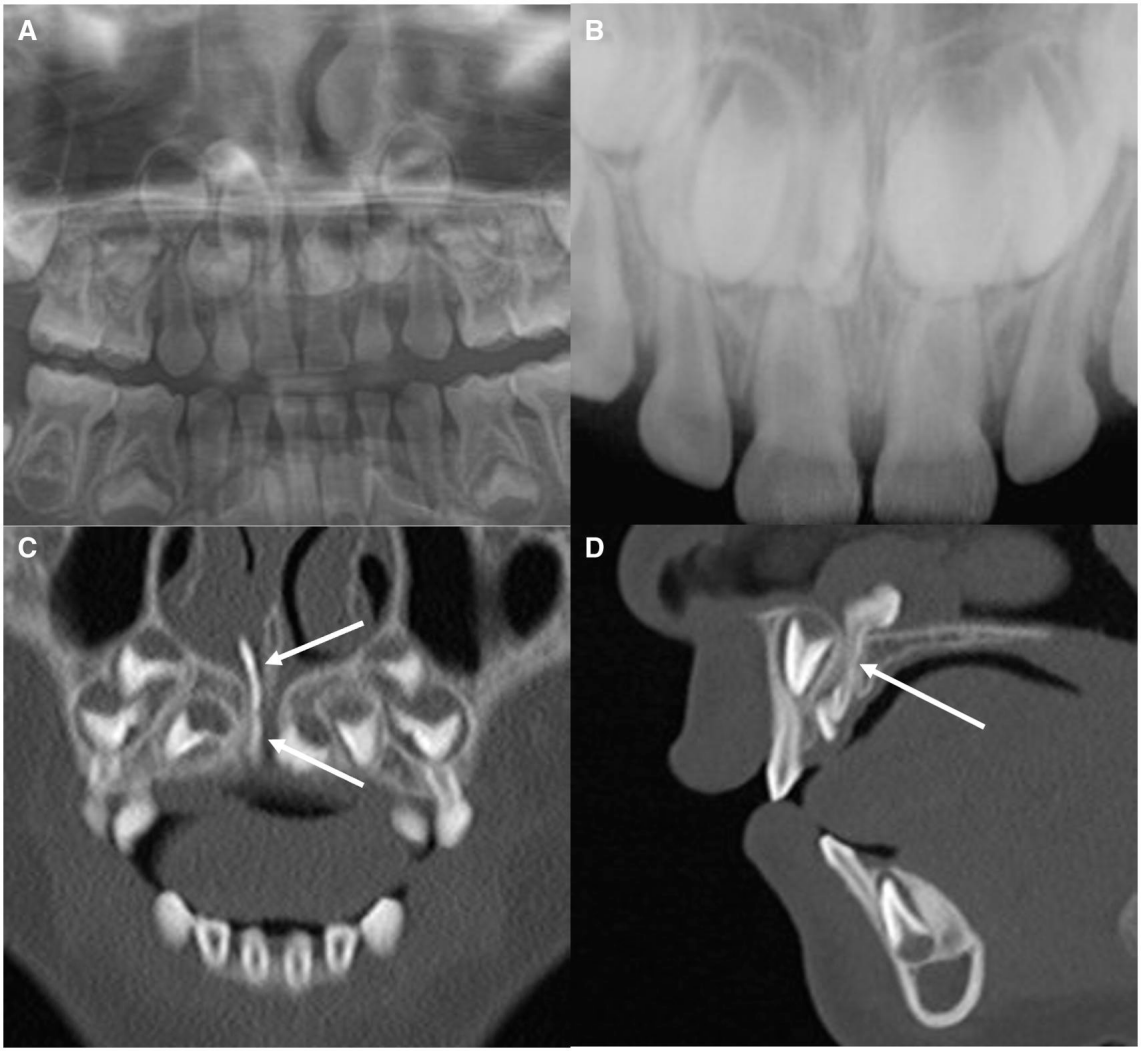
Representative image of fusion in case 14. (A, B) Cropped panoramic and occlusal radiographs, respectively. (C, D) Cropped MDCT images (coronal and sagittal views, respectively). Note the fusion of two crowns of mesiodens within the canal (D, arrow). The presence of mesiodens within the canal and the malformations is not exactly detected on panoramic and occlusal radiographs.

**Figure 4.**
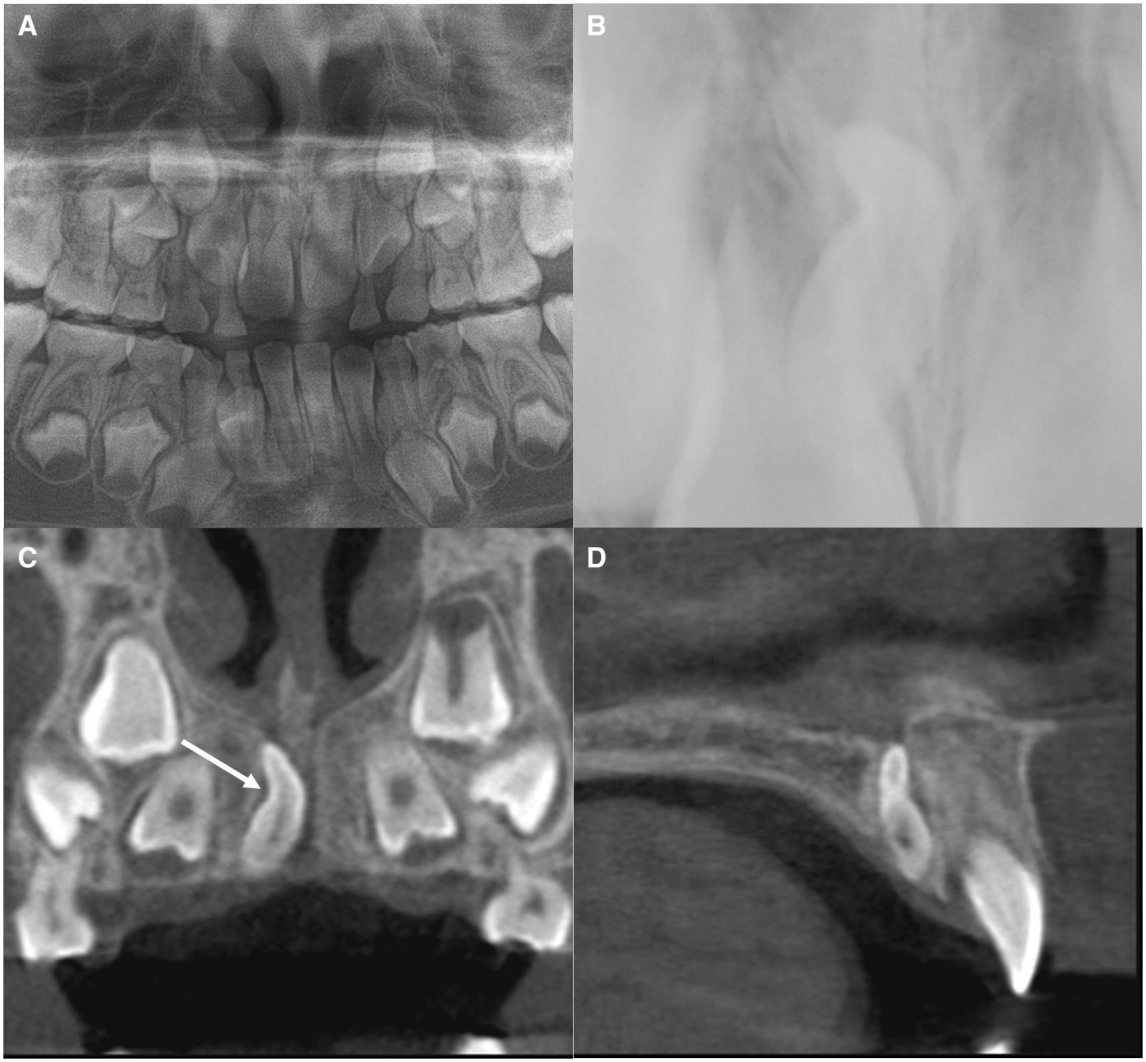
Dilaceration in the crown region in case 27. (A, B) Cropped panoramic and occlusal radiographs, respectively. (C, D) Cropped CBCT images (coronal and sagittal views, respectively). The arrow indicates the dilaceration in the crown region.

Number, direction of impaction, size, shape, and malformation (elongation, fusion, dilaceration) were analysed using the CT images. Mesiodens were counted based on the number of crowns that included an enamel portion. For example, if two crowns were observed and the roots were fused, they were defined as two mesiodens that had fused. Length was measured as the length of the long axis connecting the incisal tip to the root apex. If severe dilaceration like bending was observed, the lengths of two long axes were separately measured and the added value recorded. Width was recorded as the maximum width in the perpendicular to the long axis. Morphological variations such as elongation, fusion, and dilaceration were assessed. Mesiodens longer than the average length (13 mm) was classified as elongation (Figure 2).^15^ In addition, the suspected elongated portion within the tooth (crown or root) was recorded, with reference to the crown/root ratio. “Fusion” was defined as the connection between two crowns (Figure 3). Any deviation along the long axis of the tooth, linking the crown and root tip, was tagged as “dilaceration,” and the deviated part was recorded (Figure 4). To supplement our analysis, we determined whether it is possible to detect that the mesiodens was in the nasopalatine canal on panoramic radiograph.

## Results

Of the 32 patients with mesiodens in the nasopalatine canal, 23 were men and 9 were women. The patients’ demographic and general clinical data are presented in Table 1. The patients’ mean age at the time of diagnosis was 18 (range, 3-57) years. All patients presented no symptoms associated with the mesiodens within the nasopalatine canal, so these anomalies were incidentally discovered during panoramic examinations, leading to referrals for extractions.

**Table 1.** Summary of the clinical and radiographic findings of 34 mesiodens in the nasopalatine canal.

Patient No.	Age (year) /sex	PMH	No. of mesiodens (No. of mesiodens within the NPC)	Direction	Length (mm)	Width (mm)	Elongation	Fusion	Dilaceration	Detection of mesiodens within the NPC using panoramic radiographs	Extraction	Complication
1	13/M	n/s	1(1)	Inverted	14.64	4.4	O(C)	X	O(Ce)	X	O	X
2	21/M	n/s	2(1)	Inverted	15.37	6.09	O(C)	X	X	X	Unknown	Unknown
3	8/M	Epilepsy	1(1)	Normal	8.11	4.18	X	X	X	X	O	X
4	32/F	n/s	1(1)	Inverted	10.94	5.17	X	X	X	X	Unknown	Unknown
5	11/F	n/s	1(1)	Inverted	8.62	3.89	X	X	X	X	O	X
6	8/M	n/s	3(2)	Normal+Normal	21.13	8.26	n/a	O	n/a	X	O	X
7	6/M	n/s	2(1)	Inverted	13.85	3.82	O(C)	X	O(Ce)	O	O	X
8	9/M	n/s	1(1)	Inverted	20.81	4.0	O(C)	X	O(Ce)	X	O	Unknown
9	29/M	n/s	1(1)	Inverted	12.49	5.64	X	X	X	X	Unknown	Unknown
10	9/M	Dust, nut allergy	1(1)	Inverted	11.31	5.74	X	X	X	X	O	X
11	7/M	Rhinitis	3(2)	Normal+Inverted	21.28	9.58	n/a	O	n/a	X	O	X
12	15/M	Developmental disorder (ability)	1(1)	Inverted	13.78	5.35	O(C)	X	O(C)	X	O	X
13	11/M	n/s	2(1)	Inverted	18.6	3.5	O(C)	X	X	X	Unknown	Unknown
14	3/M	n/s	3(2)	Normal+ Inverted	22.24	9.32	n/a	O	n/a	O	O	X
15	57/F	Hypothyroidism, spasmodic dysphonia, hyperlipidaemia	1(1)	Inverted	11.26	5.76	X	X	X	X	Unknown	Unknown
16	4/M	Orchiopexy	1(1)	Inverted	13.92	7.91	O(C)	X	X	X	O	Remnant
17	8/F	Pneumonia surgery	1(1)	Inverted	16.99	4.94	O(C)	X	O(Ce)	O	O	X
18	25/M	Craniopharyngioma operation (hormonal agent)	1(1)	Inverted	16.41	4.94	O(C)	X	O(R)	X	Unknown	Unknown
19	24/F	n/s	2(1)	Inverted	12.44	4.95	X	X	O(C)	X	O	X
20	30/M	n/s	2(2)	Inverted	14.15	5.09	O(C)	X	O(C)	X	X	Unknown
				Inverted	11.38	5.79	X	X	X	X		
21	23/M	n/s	1(1)	Inverted	13.08	3.28	O(C)	X	O(Ce)	X	X	Unknown
22	24/M	n/s	1(1)	Inverted	17.7	5.02	O(C)	X	O(Ce)	X*	O	X
23	21/F	n/s	1(1)	Inverted	10.78	4.51	X	X	X	X	Unknown	Unknown
24	26/M	n/s	1(1)	Inverted	9.88	4.71	X	X	X	X*	X	X
25	20/F	n/s	1(1)	Normal	8.64	3.98	X	X	X	X*	O	X
26	6/M	n/s	2(1)	Inverted	16.56	2.83	O(C, R)	X	O(Ce)	O	O	Remnant
27	7/M	n/s	1(1)	Inverted	13.86	4.35	O(C)	X	O(C)	X	O	X
28	13/M	n/s	1(1)	Inverted	14.7	4.35	O(C)	X	X	O	O	X
29	7/M	n/s	1(1)	Inverted	13.7	5.94	O(C)	X	X	X	O	X
30	52/F	HTN, osteoporosis, kidney transplantation (immunosuppressive drug)	1(1)	Inverted	7.93	5.43	X	X	X	X	O	X
31	26/F	n/s	3(2)	Normal+Inverted	16.21	3.6	n/a	O	n/a	X	O	X
32	8/M	n/s	2(2)	Normal	11.44	4.47	X	X	X	X	O	X
				Inverted	10.45	4.06	X	X	X			

Abbreviations: No. = number, PMH = past medical history, NPC = nasopalatine canal, HTN = hypertension, X* = cannot detect mesiodens itself in panoramic radiographs, C = crown, R = root, Ce = cervical.

A total of 38 mesiodens within the nasopalatine canal were found in 32 patients. Four patients had fused mesiodens, and two patients had two separate supernumerary teeth located within the nasopalatine canal. Eight patients (21.1%) had mesiodens impacted in the normal direction, whereas the vast majority, 30 patients (78.9%), had mesiodens impacted in an inverted direction.

The morphological characteristics of the mesiodens are also presented in Table 1. A remarkable feature was thin, elongated teeth within the canal. The average length of the mesiodens was 13.13 mm (range, 7.93-20.81 mm) of the 30 mesiodens (except 4 fused teeth), and 16 (53.33%) exceeded the average length of 13.0 mm, categorizing them as elongated. The average width of the mesiodens was 4.80 mm (range, 2.83-7.91 mm). Except one mesiodens, all (96.7%) had a width diameter below the average of 6.8 mm. For the 16 elongated mesiodens, the elongation appeared to be in the crown (15/16, 93.7%) (Figure 2) and both the crown and root (1/16, 6.2%). As other morphological variations, four patients (11.76%) had fusion and 12 (40%) exhibited dilaceration. Among the latter, dilaceration was observed at the cervical region in seven patients (58.3%), crown region in four (33.3%, Figure 4), and root region in one (8.3%). In total, a large number of patients (21/34, 61.7%) presented malformations, such as elongation, fusion, and dilaceration.

Excluding 7 patients without records, 22 (23/25, 88%) underwent extraction, of whom only 2 experienced complications. They had asymptomatic tooth remnants but did not receive additional treatment. These two patients who had complications followed by mesiodens extraction had malformed mesiodens (one had elongated mesiodens, and the other had elongated and fused mesiodens). When panoramic radiographs were used, the presence of mesiodens within the nasopalatine canal was detected in only 15.6% of the patients (5/32).

## Discussion

This study presented uncommon cases of mesiodens located within the nasopalatine canal, and their morphological variations were critically analysed via CT. Of the 32 patients examined, a majority (61.76%) had malformations, such as elongation, fusion, and dilaceration. Notably, surgical interventions for mesiodens extraction did not result in anticipated complications. However, in two patients, the tooth remnants persisted postoperatively without any symptom.

To the best of our knowledge, studies that investigated the association between mesiodens and the nasopalatine canal, especially when not associated with a pathology such as a nasopalatine duct cyst, are scarce.^16^ Mossaz et al reported that 20.5% of mesiodens are proximate to the cortical bone of the nasal floor, whereas 49% are related to the nasopalatine canal.^10^

Interestingly, mesiodens extending into the nasopalatine canal often exhibit abnormal shapes. Though a few studies have investigated mesiodens within the canal, none have included as many as 32 cases or explored the associated morphological variations in our study.^10^ Only two case reports regarding mesiodens within the canal were available.^17^^,^^18^ One reported a conical mesiodens with a typical appearance but no specific symptom.^17^ Another report presented a male patient with a 2-year history of right-sided nasal obstruction and occasionally blood-stained discharge who had an elongated and fused mesiodens extending to the inferior nasal cavity.^18^ In a notable study, mesiodens measurements widely varied, with lengths ranging from 3.9 to 26.0 mm (average, 13.0 mm)—approximately half the average length of standard maxillary central incisors, which is 23.5 mm.^15^ In terms of width, the measurements varied from 3.1 to 19.7 mm (average, 6.8 mm).^19^ In the present study, the average length was greater and the average width was smaller compared with this, resulting in a predominantly thin and elongated morphology. The findings from this observational study add significant knowledge in the development of mesiodens within the nasopalatine canal. While the aetiology of midline supernumerary teeth remains unclear, multiple theories abound.

Odontogenesis results in the formation of unique shapes for the crown and root of each type of tooth.^19^ Molecular patterning may influence dental evolution through differences in gene expressions associated with morphological variations. Signalling pathway can have an impact on every stage of tooth development, starting as early as the 6th to the 7th week of gestation (and even earlier, around day 22 when considering neural crest cells) and continuing until the age of 20-25.^19^ The nasopalatine canal also develops at the same stage.^20^ Therefore, if a developing tooth bud enters the nasopalatine canal through the epithelial–mesenchymal signal pathway, the morphology of mesiodens might be adapted to the narrow and elongated shape of the canal.

The timing and necessity of mesiodens extraction are subjects of ongoing debate from the perspective of technical difficulty of extraction, occlusal complications, and patient cooperation. Primosch et al have suggested waiting for intervention until the neighbouring teeth have almost completed root development until the patient reaches the age of 8-10.^21^ In 75% of cases, the incisor spontaneously erupts once the mesiodens has been removed.^14^^,^^22^ Extraction during the early mixed dentition stage enables normal eruptive forces to promote spontaneous eruption of the permanent central incisors following the extraction.^11^^,^^12^^,^^14^

Given the proximity of mesiodens to permanent teeth and potential complications, an accurate diagnosis is important before opting for extraction. In general, extraction without retained roots can be challenging when morphological variations exist in the teeth. The same applies to mesiodens, and when such situations involve the nasopalatine canal, there is a higher likelihood of disturbing structures that include the nerves and blood vessels, raising concerns regarding complications.

Therefore, we hypothesized that mesiodens within the nasopalatine canal would exhibit a high degree of morphological variation and that this would increase post-extraction complications such as tooth remnant or neurogenic, vascular complications. However, object to our hypothesis, neurogenic or vascular complications associated with the nasopalatine canal were minimal at the time of suture removal (1 week later post-operation). In another study, there were very few postoperative complications associated with mesiodens extraction, and these complications were related to pulp necrosis or delayed eruption of adjacent permanent teeth.^7^^,^^23^

Various surgeries that invade the nasopalatine canal, such as implant fixture placement, have fewer postoperative complications than expected. Anastomosis of the nasopalatine nerve and sphenopalatine artery with the greater palatine nerve and artery enables immediate revascularization and gradual reinnervation of the maxillary anterior region. Furthermore, the pulp and periodontal tissue of the anterior maxillary teeth are innervated by the anterior superior alveolar nerve rather than the nasopalatine nerve. Thus, surgeries encroach upon the nasopalatine canal do not seemed to cause the significant neurosensory disturbance of maxilla.^24^ However, in rare cases, neurosensory changes may occur, and the risk varies depending on the location of the impacted tooth that requires extraction.^25^

Although excluded in our study, in cases where a widened canal was diagnosed as a dentigerous cyst postoperatively, postoperative neurologic disturbances occurred. While the occurrence of neurologic complications is relatively rare, when a mesiodens is located within the nasopalatine canal, proper informed consent and adequate preoperative assessment on the morphological variations of mesiodens and their association with the canal are imperative.

The complication that occurred in two patients was tooth remnant after extraction; these two patients had elongated mesiodens and elongated, fused mesiodens, respectively. Although the number is small, morphological variations, including elongation and fusion, are more likely to cause complications such as tooth fracture during extraction. Therefore, special attention should be given to the treatment of mesiodens with an atypical morphology. For extraction planning, it is necessary to assess the tooth position and morphology and to formulate the surgical approach and method through imaging. Typically, a panoramic image is initially taken to evaluate the overall tooth count, developmental stage, and position. However, as in the present study, determining the presence of mesiodens within the nasopalatine canal was challenging with panoramic imaging alone. Thus, additional imaging assessment methods, particularly the use of three-dimensional CT images that can evaluate the position, shape, and potential impact on adjacent structures and even particular morphology, need to be established.

This study had some limitations. As a retrospective study, data were based on written patient records on surgical treatment and postoperative symptoms. Thus, some details such as specific surgical methods or minor complications might have been ignored. Furthermore, the inherent morphological variability of supernumerary teeth makes it difficult to define “normal” or “average” standards; hence, description of certain assessments is more subjective. Further research that includes larger sample sizes and investigates the morphological differences between standard mesiodens and those within the nasopalatine canal is required. Further study on the detailed mechanisms of tooth development within the nasopalatine canal is also necessary.

## Conclusion

In summary, while mesiodens are quite common in paediatric dental presentations, those within the nasopalatine canal are rarely reported. This study, which includes a relatively significant sample size, highlights the importance of recognizing and addressing the distinct morphological variations of mesiodens located within the canal. It is crucial to ensure detailed three-dimensional imaging evaluations before surgical interventions to optimize patient outcomes and reduce potential complications.

## References

[1] Mason C , Azam N, Holt RD, Rule DC. A retrospective study of unerupted maxillary incisors associated with supernumerary teeth. Br J Oral Maxillofac Surg. 2000;38(1):62-65. 10783451 10.1054/bjom.1999.0210

[2] Esenlik E , Sayin MO, Atilla AO, Ozen T, Altun C, Başak F. Supernumerary teeth in a Turkish population. Am J Orthod Dentofacial Orthop. 2009;136(6):848-852. 19962608 10.1016/j.ajodo.2007.10.055

[3] Demiriz L , Durmuşlar MC, Mısır AF. Prevalence and characteristics of supernumerary teeth: a survey on 7348 people. J Int Soc Prev Community Dent. 2015;5(Suppl 1):S39-43. 25984466 10.4103/2231-0762.156151 PMC4428018

[4] Celikoglu M , Kamak H, Oktay H. Prevalence and characteristics of supernumerary teeth in a non-syndrome Turkish population: associated pathologies and proposed treatment. Med Oral Patol Oral Cir Bucal. 2010;15(4):e575-e578. 20173719 10.4317/medoral.15.e575

[5] Van Buggenhout G , Bailleul-Forestier I. Mesiodens. Eur J Med Genet. 2008;51(2):178-181. 18262485 10.1016/j.ejmg.2007.12.006

[6] Primosch RE. Anterior supernumerary teeth-assessment and surgical intervention in children. Pediatr Dent. 1981;3(2):204-215. 6945564

[7] Asaumi JI , Shibata Y, Yanagi Y, et al Radiographic examination of mesiodens and their associated complications. Dentomaxillofac Radiol. 2004;33(2):125-127. 15314006 10.1259/dmfr/68039278

[8] Zhang LL , Yang R, Zhang L, Li W, MacDonald-Jankowski D, Poh CF. Dentigerous cyst: a retrospective clinicopathological analysis of 2082 dentigerous cysts in British Columbia, Canada. Int J Oral Maxillofac Surg. 2010;39(9):878-882. 20605411 10.1016/j.ijom.2010.04.048

[9] Lustmann J , Bodner L. *Dentiger0us* cysts associated with supernumerary teeth. Int J Oral Maxillofac Surg. 1988;17(2):100-102. 3133415 10.1016/s0901-5027(88)80159-0

[10] Garvey MT , Barry HJ, Blake M. Supernumerary teeth-an overview of classification, diagnosis and management. J Can Dent Assoc. 1999;65(11):612-616. 10658390

[11] Witsenburg B , Boering G. Eruption of impacted permanent upper incisors after removal of supernumerary teeth. Int J Oral Surg. 1981;10(6):423-431. 6809665 10.1016/s0300-9785(81)80079-8

[12] Solares R. The complications of late diagnosis of anterior supernumerary teeth: case report. ASDC J Dent Child. 1990;57(3):209-211. 2345215

[13] Yague-Garcia J , Berini-Aytes L, Gay-Escoda C. Multiple supernumerary teeth not associated with complex syndromes: a retrospective study. Med Oral Patol Oral Cir Bucal. 2009;14(7):E331-6. 19300360

[14] Tay F , Pang A, Yuen S. Unerupted maxillary anterior supernumerary teeth: report of 204 cases. ASDC J Dent Child. 1984;51(4):289-294. 6590583

[15] Kang E , Choi N, Kim S. Three dimensional analysis of maxillary mesiodens using dental CBCT and relationship between the mesiodens and diastema. J Korean Acad Pediatr Dent. 2013;40(4):260-267.

[16] Mossaz J , Kloukos D, Pandis N, Suter VG, Katsaros C, Bornstein MM. Morphologic characteristics, location, and associated complications of maxillary and mandibular supernumerary teeth as evaluated using cone beam computed tomography. Eur J Orthod. 2014;36(6):708-718. 24385409 10.1093/ejo/cjt101

[17] Aoun G , Nasseh I. Mesiodens within the nasopalatine canal: an exceptional entity. Clin Pract. 2016;6(4):903. 28174622 10.4081/cp.2016.903 PMC5294929

[18] Misirovs R , Kanodia AK, McDonald C, Green R. Supernumerary tooth in nasopalatine canal: a rare cause of septal cartilage collapse. BMJ Case Rep. 2021;14(9):e245103. 10.1136/bcr-2021-245103 PMC8458351 34548301

[19] Bloch-Zupan A , Sedano HO, Scully C, Odontogenesis, anomalies and genetics In: Bloch-Zupan A, Scully C, Sedano HO, eds. Dento/Oro/Craniofacial Anomalies and Genetics. Elsevier science; 2012:1-8.

[20] Kim JH , Oka K, Jin ZW, et al Fetal development of the incisive canal, especially of the delayed closure due to the nasopalatine duct: a study using serial sections of human fetuses. Anat Rec (Hoboken). 2017;300(6):1093-1103. 27860365 10.1002/ar.23521

[21] Shih WY , Hsieh CY, Tsai TP. Clinical evaluation of the timing of mesiodens removal. J Chin Med Assoc. 2016;79(6):345-350. 27090104 10.1016/j.jcma.2015.10.013

[22] Howard RD. The unerupted incisor. A study of the postoperative eruptive history of incisors delayed in their eruption by supernumerary teeth. Dent Pract Dent Rec. 1967;17(9):332-341. 5228619

[23] Alacam A , Bani M. Mesiodens as a risk factor in treatment of trauma cases. Dent Traumatol. 2009;25(2):e25-e31. 19290890 10.1111/j.1600-9657.2008.00734.x

[24] Spin-Neto R , Bedran TB, de Paula WN, de Freitas RM, de Oliveira Ramalho LT, Marcantonio E. Jr. Incisive canal deflation for correct implant placement: case report. Implant Dent. 2009;18(6):473-479. 20009600 10.1097/ID.0b013e3181bd0c7c

[25] de Mello JS , Faot F, Correa G, Chagas Júnior OL. Success rate and complications associated with dental implants in the incisive canal region: a systematic review. Int J Oral Maxillofac Surg. 2017;46(12):1584-1591. 28552441 10.1016/j.ijom.2017.05.002

